# Breed composition does not influence the performance of Holstein-Gyr crossbred as oocyte donors for OPU/IVP

**DOI:** 10.21451/1984-3143-2017-AR978

**Published:** 2018-08-16

**Authors:** Clara Ana Santo Monteiro, Helena Fabiana Reis de Almeida Saraiva, Gabriela Ramos Leal, Agostinho Jorge dos Reis Camargo, Raquel Varella Serapião, Ana Maria Reis Ferreira, André Luís Rios Rodrigues, Luiz Altamiro Garcia Nogueira, Clara Slade Oliveira

**Affiliations:** 1 Federal Fluminense University (UFF), , , .; 2 , , .; 3 Embrapa Dairy Cattle, Laboratory of Animal Reproduction, Santa Monica Experimental Field (LRA-CESM), Valença, , .

**Keywords:** Crossbred, *Girolando*, Oocyte donor, OPU

## Abstract

Holstein-Gyr crossbred cattle are strategic for dairy systems in tropical countries, since they combine milk yield genetics with adaptability to tropical climate. However, Holstein (*Bos taurus*) and Gyr (*Bos indicus*) breeds present remarkable differences regarding reproductive physiology. Brazil stands out as the world’s largest user of embryo *in vitro* production (IVP) in bovine, and the use of this technique is increasing in dairy systems. As Holstein-Gyr crossbreds are important oocyte donors for IVP, the present work aimed at investigating whether increased Gyr or Holstein breed composition influences donor’s performance. Sixteen Holstein-Gyr crossbred females presenting increased (HG, 71.4 to 87.5% Holstein; n = 9) or decreased (GH, 40.2 to 46.6% Holstein; n = 7) Holstein composition were submitted to three ovum pick up (OPU) sessions. We observed similar (P = 0.2946) antral follicle count between HG and GH donors (24.8 ± 3.2 *vs* 29.4 ± 2.8 respectively; mean ± SEM). Groups also display similar morphological oocyte grading (Grade I: 0.1 ± 0.1 *vs* 0.1 ± 0.1 – P = 0.9680; Grade II: 0.9 ± 0.5 *vs* 1.9 ± 0.5 – P = 0.1942; Grade III, 4.0 ± 1.2 *vs* 7.2 ± 1.4 – P = 0.1047, HG *vs* GH respectively; mean ± SEM). Additionally, the proportion of viable oocyte was similar between HG and GH groups (27.8% *vs* 31.9%, respectively, P = 0.3500) and oocyte lipid area fraction (6.8% *vs* 9.5%, respectively; P = 0.1539). Our results indicate that the individual variation has more influence than breed composition of crossbred oocyte donors.

## Introduction

Embryo *in vitro* production (IVP) is an important reproductive biotechnology for livestock. Cattle IVP allows the rapid dissemination of valuable genetic traits of herds. South America accounted for 66.9% of world’s OPU/IVP bovine embryo production, being 95.2% of those produced in Brazil ( Chair, 2016 ). The use of this biotechnology has grown in dairy systems, in which oocytes can be collected from lactating and pregnant animals, and fertilized with frozen sperm from bulls with high estimated breeding value.

Holstein-Gyr crossbred herds are essential to dairy systems in tropical countries, being responsible for nearly 80% of Brazilian milk production ( Silva *et al*., 2013 ), because combine high milk yield and adaptability to tropical conditions. Purebred Holstein (*Bos taurus*) and Gyr (*Bos indicus*) donors are used to produce F1 females (1/2 Holstein, 50%), which are required to produce 3/4 (75% Holstein) and 5/8 (62.5% Holstein), also in great demand.

The subspecies *Bos taurus* and *Bos indicus* present several differences in reproductive physiology, including differences in ovarian follicular dynamics, number of follicular waves, follicular growth rate and maximum size of the dominant follicle ( Figueiredo *et al*., 1997 ; Bó *et al*., 2003 ). Thus, distinct hormonal treatments protocols are required for artificial insemination and superovulation ( Baruselli *et al*., 2011 ).

Reports show a greater lipid content in *Bos taurus* oocytes ( Ordoñez-Leon *et al*., 2014 ) and embryos ( Visintin *et al*., 2002 ). Intracellular lipid content is related to oocyte competence and embryonic development, being an energy reserve necessary to supply the metabolic demands of gamete and further embryo ( McEvoy *et al*., 2000 ; Jeong *et al*., 2009 ; Silva-Santos *et al*., 2014 ). Moreover, studies have shown that oocytes and embryos with high lipid content have greater sensitivity to cryopreservation ( Abe *et al*., 2002 ; Sudano *et al*., 2011 ). Such differences highlight importance of a morphological characterization of crossbred female oocytes in order to standardize *in vitro* production and cryopreservation protocols suitable for specific crossbred composition.

Since *Bos taurus* and *Bos indicus* present such remarkable differences, breed composition could influence Holstein-Gyr crossbred performance as oocyte donors for OPU/IVP. Therefore, it is critical to understand physiological differences and possible limitations of oocytes from donors with different breed compositions.

The aim of this study was to compare Holstein-Gyr crossbred females with increased Gyr or Holstein breed composition with regard to their performance as oocyte donors for OPU/IVP.

## Materials and Methods

### Animals

We studied 16 Holstein-Gyr crossbred donors, non-lactating, with an average age of 4 to 9 years and weight between 380 - 410 kg that presented cyclical ovarian activity. Animals were kept on pasture with water and mineral supplementation available ad libitum. All procedures were approved by Embrapa Dairy Cattle Committee of Ethics in Animal Use (13/2013 protocol). Experiments took place at the Embrapa Dairy Cattle, Laboratory of Animal Reproduction, Santa Monica Experimental Field (LRA-CESM), located in Valença, RJ.

### Study design

Donors were grouped according to their breed composition as HG (Holstein-Gyr crossbred presenting increased Holstein composition – 71.4 to 87.5% Holstein; n = 9) and GH (Holstein-Gyr crossbred presenting increased Gyr composition - 40.2 to 46.6% Holstein; n = 7). Three ovum pick up (OPU) sessions were carried out, and performance of each donor was recorded, regarding (1) antral follicle count; (2) morphological oocyte grading (Grade I, Grade II, Grade III and total); (3) proportion of viable oocytes; and (4) oocyte lipid area fraction. Donors were randomly submitted to OPU, without hormonal stimulation, with a minimum of 15 days interval between sessions.

### Ovum pick-up

Oocytes were recovered by OPU. Donors were submitted to epidural anesthesia (lidocaine hydrochloride 2%) and were kept in a cattle chute throughout procedure. A transvaginal device guiding both needle and a 7.5 MHz convex transducer coupled to ultrasound equipment (Mindray DP2200) were used. Before follicle aspiration, all antral follicles were counted. Then, follicles between 2 and 8 mm were punctured with 18 gauge x 50 mm needles and the follicular fluid was aspirated through a vacuum system (90 mmHg). Follicular fluid collected from each donor was set individually in 50 ml tube containing Dulbecco’s phosphate-buffered saline (DPBS) supplemented with 10 UI/ml heparin and 10% fetal calf serum (FCS) solution, at 37°C.

### Oocyte recovery

DPBS solution containing follicular fluid was transferred into filters for oocyte collection and washed with DPBS heated at 37°C. Fluid was transferred to Petri dish (100 x 20 mm) for *cumulus* oocyte complexes (COCs) tracking using a stereomicroscope (Olympus SZ40). COCs were washed in TCM-199 buffered with HEPES (Gibco BRL, Grand Island, NY) supplemented with 1.0 mM sodium pyruvate (Gibco BRL, Grand Island, NY) and 100 UI penicillin and 0.1mg/ml streptomycin. Morphologic oocyte grading was performed as follows: grade I, oocytes presenting more than 3 *cumulus* cells layers and homogenous cytoplasm; grade II, oocytes presenting less than 3 *cumulus* cells layers and cytoplasm with minor granules; grade III, oocytes discontinuously surrounded by *cumulus* cells and/or cytoplasm displaying major granules; non-viable, oocytes without *cumulus* cells, degenerated or expanded.

### Lipid area fraction

For analysis of lipid area fraction, Oil Red staining (Oil Red O solution, Sigma) was performed. Briefly, immature oocytes were denuded in 2 mg/ml hyaluronidase solution, fixed in 4% paraformaldehyde (PFA) solution for 30 to 40 min, and stored at 4°C. Then structures were washed in 50% ethanol solution for 2 min, and stained in oil red solution for 10 min, followed by 3 washes in 50% ethanol solution for 5 min each, and finally washed in distilled water 5 min. Stained structures were mounted on slides for analysis. Images of each structure were captured (Alltion ABM200 series; Sony Cyber Shot 7.2 mega pixels) and evaluated by Image J software as oocyte area fraction composed by lipids. All structures were stained, photographed and analyzed at the same day.

### Statistical analysis

Antral follicle count, morphological oocyte grading (Grade I, Grade II, Grade III) and lipid area fraction were compared between groups using Unpaired T test or Mann-Whitney test, for non-parametrical data. Proportions of viable oocytes rates were compared between groups using Fisher exact test. Statistical analysis was performed using GraphPad INSTAT 3 program, considered significance level of 5%.

## Results

### Breed composition does not affect antral follicle count, morphological oocyte grading and viable oocyte recovery of Holstein-Gyr crossbred

We found similar antral follicle count between (HG and GH groups; HG vs GH respectively; Table 1 ).

**Table 1:** Holstein-Gyr crossbred presenting increased (HG) and decreased (GH) Holstein breed composition performance as oocyte donor for OPU/IVP. For analysis, 16 Holstein-Gyr crossbred females were subjected to 3 ovum pick up (OPU) sections.

Groups	Antral follicle count (Mean ± SEM)	Morphological oocyte grading (Mean ± SEM)			Viable oocyte (%)	Lipid area fraction (Mean ± SEM)
		GI	GII	GIII		
HG	24.8 ± 3.2	0.1^ab^ ± 0.1	0.9^b^ ± 0.5	4.0^c^ ± 1.2	27.8	6.8 ± 1.1
GH	29.4 ± 2.8	0.1^a^ ± 0.1	1.9^b^ ± 0.5	7.2^c^ ± 1.4	31.9	9.5 ± 1.4

Superscript letters denote statistical difference (P < 0.05).

From a total of 468 oocytes obtained in 3 replicates, HG and GH groups showed similar distribution among GI (GI: 0.1^ab^ ± 0.1 *vs* 0.1^a^ ± 0.1 – P = 0.9680), GII (0.9^b^ ± 0.5 *vs* 1.9^ b^ ± 0.5 – P = 0.1942) and GIII (4.0^c^ ± 1.2 *vs* 7.2^c^ ± 1.4 – P = 0.1047; HG *vs* GH respectively; mean ± SEM). However, the comparison between mean number of GI, GII and GIII oocytes within HG and GH groups, demonstrated that in the HG group, GI and GII oocytes means were similar (P = 0.0336), and these were lower (GI *vs* GIII: P = 0.0039 and GII *vs* GIII: P = 0.0336) than GIII (GI = GII < GIII), whereas in the GH group, GI oocyte number was lower (P = 0.0147) than GII and (P = 0.0009) GIII; and GII was lower (P = 0.0028) than GIII (GI < GII < GIII; Table 1 ).

Also, HG and GH groups presented similar (P = 0.3500) proportions of viable oocytes (27.8% *vs* 31.9%; Table 1 ).

### Breed composition does not influence oocyte lipid content

From the total oocytes recovered in 2 out of 3 replicates, 60 viable oocytes were evaluated for lipid area fraction (HG, n = 24; GH, n = 36). Groups displayed similar lipid content means (P = 0.1539; Table 1 ). This corresponds to oocyte area fraction (mean ± SEM) of 6.8% ± 1.1 in HG group and 9.8% ± 1.4 in GH group.

## Discussion

In the present study, we compared Holstein-Gyr crossbred females with increased Holstein (HG) or Gyr (GH) breed composition regarding their performance as oocyte donors for OPU/IVP.

Regarding donor’s performance in OPU, it is known that *Bos indicus* and *Bos taurus* breeds display significant differences in ovarian characteristics, such as size and number of visible ovarian follicles. *Bos indicus* exhibit a larger number of follicular growth waves (approximately ≈ 3)leading to a higher number of visible ovarian follicles of a relatively smaller size ( Viana *et al*., 2004 ), while *Bos taurus,* with fewer follicular growth waves (approximately ≈ 2), present a smaller number of visible ovarian follicles, which have a relatively larger size. Additionally, *Bos indicus* donors are widely known for exhibit grater follicle population compared to *Bos taurus* donors (Pontes *et al*., 2010). Therefore, it is most likely to assume that the crossbred donors with major Gyr or Holstein breed composition could preserve characteristics of their primary breed composition. However, according to our results, breed composition does not influence ovarian follicular count under the present conditions.

Oocyte quality is crucial for IVP success and we comparedmorphological parameters in order to assess breed composition influence on oocyte quality. Regardless breed composition, proportion of viable oocytes recovered and distribution into GI, GII and GIII oocytes groups was similar. We observed that these characteristics have a great individual variation which may have masked possible effects of donors breed composition.

Additionally, breed composition did not affect lipid content considering same experimental conditions for both groups, despite the reported differences in lipid content ( Ordoñez-Leon *et al*., 2014 ) and cryotolerance ( Sudano *et al*., 2011 ) between *Bos taurus* and *Bos indicus* samples. This seems to indicate that the variation of Holstein composition applied in the present study is not sufficient to influence this characteristic.

Our results indicated that differences in Holstein composition as high as 31 to 40% did not interfere with Holstein-Gyr crossbred performance at OPU/IVP sessions. We acknowledge that difference between groups is not very breadth and a limited number of observations, however Bos indicus x Bos taurus crossbred reproductive traits are subjected to a great individual variation ( Bridges *et al*., 2005 ).

Therefore, in our conditions, individual variation seems to be more important than the degree of Holstein composition when selecting Holstein-Gyr crossbred as oocyte donors for IVP.
